# Ginsenoside Rh3 activates Nrf2 signaling and protects endometrial cells from oxygen and glucose deprivation-reoxygenation

**DOI:** 10.18632/aging.103009

**Published:** 2020-04-07

**Authors:** Xiu-mei Wang, Chang She, Quan Li, Di Zhang, Jin-xia Xu, Min-hui Li, Ping Li, Hong-bin Xu

**Affiliations:** 1 Obstetrics and Gynecology Department, Huai’an Maternal and Child Health Hospital, Huai’an, China; 2 Department of Orthopedics, the Second Affiliated Hospital of Soochow University, Suzhou, China; 3 Center of Stomatology, the Second Affiliated Hospital of Soochow University, Suzhou, China; 4 Dalian Medical University, Dalian, China; 5 Obstetrics and Gynecology Department, The Affiliated Changzhou No. 2 People’s Hospital of Nanjing Medical University, Changzhou, China; 6 Department of Radiotherapy and Oncology, Affiliated Kunshan Hospital of Jiangsu University, Suzhou, China

**Keywords:** ginsenoside Rh3, Nrf2, OGDR, oxidative injury

## Abstract

Oxygen and glucose deprivation (OGD)-reoxygenation (OGDR) induces oxidative injury to endometrial cells *in vitro*. We tested the potential effect of ginsenoside Rh3 (GRh3) in the process. Our results show that GRh3 activated Nrf2 signaling in T-HESC cells and primary murine endometrial cells. GRh3 induced Nrf2 Ser-40 phosphorylation and Keap1-Nrf2 disassociation, causing Nrf2 protein stabilization and nuclear translocation, which led to transcription and expression of antioxidant response element-dependent genes (*HO1*, *NQO1* and *GCLC*). In T-HESC cells and primary murine endometrial cells, GRh3 potently attenuated OGDR-induced reactive oxygen species production, lipid peroxidation and mitochondrial depolarization, as well as cell viability reduction and necrosis. Activation of Nrf2 is required for GRh3-induced anti-OGDR actions in endometrial cells. Nrf2 inhibition, by Nrf2 shRNA, knockout (through CRISPR-Cas9-editing) or S40T mutation, abolished GRh3-induced endometrial cell protection against OGDR. Additionally, forced activation of Nrf2, by Keap1 knockout, mimicked and nullified GRh3-induced anti-OGDR actions in T-HESC cells. Together, we conclude that GRh3 protects endometrial cells from OGDR via activation of Nrf2 signaling.

## INTRODUCTION

Postpartum hemorrhage is a common but severe complication in clinical obstetrics [1–3]. It can lead to significant endometrium ischemic injury [1–3]. When accompanied with reperfusion, ischemia will cause further oxidative damage to the endometrium [1–3]. In the clinical practice, the postpartum hemorrhage is often more likely to occur in the aged parturient. Existing studies have shown that ischemia-reperfusion will induce profound reactive oxygen species (ROS) production [4–6], oxidative stress, and lipid peroxidation as well as substantial protein damage and profound DNA injury, and eventually endometrial cell death [1–6]. Our group has been using an *in vitro* oxygen and glucose deprivation (OGD)-reoxygenation (OGDR) procedure [7, 8], that mimics ischemia-reperfusion injury to cultured endometrial cells.

Nuclear-factor-E2-related factor 2 (Nrf2) is a well-known transcription factor, promoting the transcription of multiple antioxidant genes and detoxifying enzymes. In the resting condition, Nrf2 stays in the cytoplasm, binding to its suppressor protein Kelch-like ECH-associated protein 1 (Keap1). With the help of Cul3 ubiquitin ligase complex, Keap1 will promote ubiquitination and proteasomal degradation of inactivated Nrf2. Once activated (*i.e.* by phosphorylation), Nrf2 will disassociate from Keap1, then allowing Nrf2 protein stabilization and cytosol accumulation [9–11]. Activated Nrf2 will translocate to cell nuclei, where it binds to antioxidant responsive element (ARE) [9–11]. It will then promote the transcription and subsequent expression of Nrf2-dependent genes, including *heme oxygenase-1 (HO-1)*, *NAD(P)H quinone oxidoreductase 1 (NQO1)*, γ-*glutamyl cysteine ligase catalytic subunit* (*GCLC*) [9]. Activation of Nrf2 in endometrial cells can alleviate OGDR-induced oxidative injury. Our previous study has shown that Nrf2 signaling activation by keratinocyte growth factor (KGF) efficiently protected endometrial cells from OGDR-induced oxidative injury [7].

Considering that ROS production and oxidative stress are key pathological mechanisms of clinical endometrium ischemic injury, agents that could provoke significant antioxidant response should have important therapeutic value for postpartum hemorrhage. Ginseng and its active component ginsenosides are widely-utilized and multi-functional traditional Chinese medicines (TCM) [12, 13]. The main active component of heat-processed ginseng is Rg5, which has displayed anti-cancer, anti-inflammatory, and ROS scavenging abilities at both cellular and physiological levels [14–17].

In humans, Ginseng Rg5 will be metabolized into Ginseng Rh3 (GRh3). The latter shows excellent pharmacological efficiency [18, 19]. GRh3 can exert significant cytoprotective functions [18, 19]. A recent study has shown that GRh3 activates Nrf2 signaling to protect human retinal cells from UV-induced oxidative injury [20]. Its activity in endometrial cells has not been studied thus far. The results of the present study show that GRh3 robustly activated Nrf2 signaling and protected endometrial cells from OGDR. Interestingly, the mutation in one Nrf2 phosphorylation site (S40T) could disrupt GRh3-induced Nrf2 signaling activation in endometrial cells.

## RESULTS

### GRh3 activates Nrf2 signaling cascade in T-HESC cells and primary murine endometrial cells

**Figure 1:**
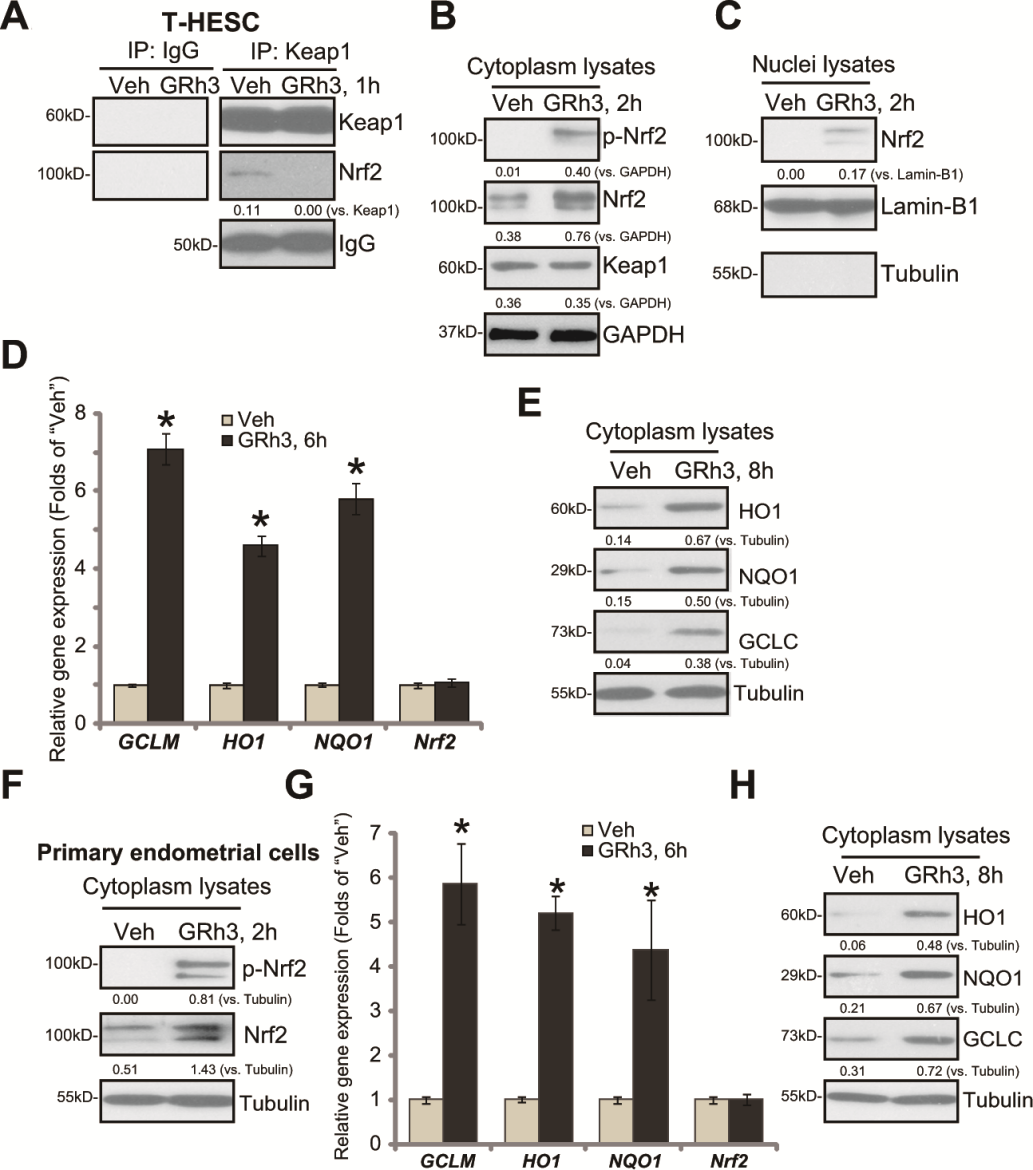
**GRh3 activates Nrf2 signaling cascade in T-HESC cells and primary murine endometrial cells.** T-HESC human endometrial cells (**A**–**E**) or the primary murine endometrial cells (**F**–**H**) were treated with GRh3 (at 10 μM) for the indicated time periods. Keap1-Nrf2 association was tested by co-immunoprecipitation assay (**A**); Expression of listed proteins (**B**, **C**, **E**, **F** and **H**) in cytosol lysates and nuclear fraction lysates was tested by Western blotting, with relative expression of listed mRNAs tested by qPCR (**D** and **G**). Expression of the listed proteins was quantified, normalizing to the loading control protein (**A**–**C**, **E**, **F** and **H**). Error bars stand for mean ± standard deviation (SD, n=5). “Veh” stands for vehicle control (PBS, same for all Figures). *p<0.05 *vs.* “Veh” (**D** and **G**). Each experiment was repeated three times with similar results obtained.

We first tested whether GRh3 could activate Nrf2 signaling in endometrial cells. Performing the co-immunoprecipitation (“Co-IP”) assay, we show that Nrf2 associated with its suppressor protein Keap1 in the untreated T-HESC human endometrial cells (Figure 1A). Significantly, following GRh3 (10 μM, based on the previous study [7]) treatment, Keap1-Nrf2 association was disrupted (Figure 1A). In the GRh3-treated T-HESC cells, GRh3 induced Nrf2 Ser-40 (S40) phosphorylation and cytosol Nrf2 protein accumulation (Figure 1B), an initial step for Nrf2 cascade activation [21–24]. Keap1 expression was, however, unchanged (Figure 1B). In response to GRh3, the accumulated Nrf2 translocated to the nuclei of T-HESC cells, causing increased Nrf2 protein in the nuclei lysates (Figure 1C).

Testing expression of the ARE-dependent genes by qPCR demonstrated that GRh3 induced expression of *HO1*, *NQO1* and *GCLC* in T-HESC cells (Figure 1D). Moreover, HO1, NQO1 and GCLC protein levels were significantly increased as well (Figure 1E). *Nrf2 mRNA* levels were however unchanged following the GRh3 stimulation (Figure 1D).

In the primary murine endometrial cells, GRh3 treatment similarly induced Nrf2 S40 phosphorylation and cytosol Nrf2 protein accumulation (Figure 1F), as well as expression of *HO1*, *NQO1* and *GCLC mRNAs* (Figure 1G) and proteins (Figure 1H). *Nrf2 mRNA* levels were again unaffected with GRh3 treatment (Figure 1G). Collectively, these results indicated that GRh3 activated Nrf2 signaling cascade in T-HESC cells and primary murine endometrial cells.

### GRh3 protects endometrial cells from OGDR

**Figure 2:**
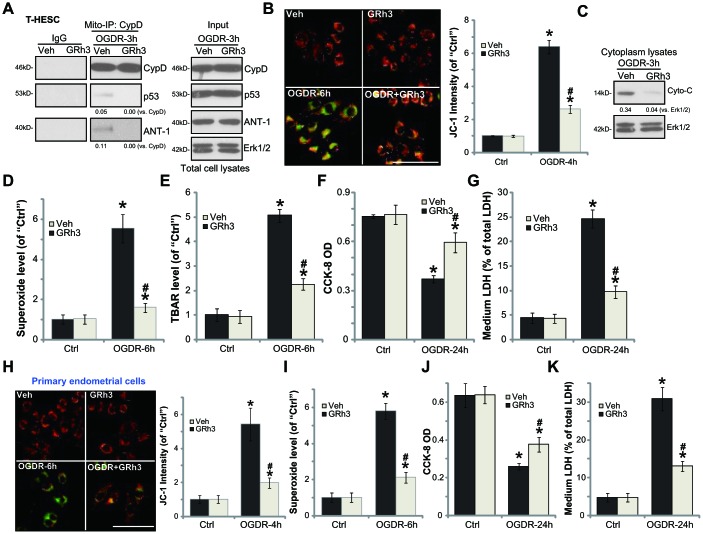
**GRh3 protects endometrial cells from OGDR.** T-HESC cells (**A**–**G**) or the primary murine endometrial cells (H-K) were pre-treated with GRh3 (10 μM, 2h pretreatment), followed by OGDR stimulation. After indicated time periods, mitochondrial CypD-p53-ANT-1 association (“Mito-IP”, **A**), mitochondrial depolarization (the JC-1 green intensity, **B** and **H**), cytochrome C (“Cyto-C”) release (**C**, testing the cytosol proteins), as well as superoxide contents (**D** and **I**) and lipid peroxidation (TBAR assay, **E**) were tested; Cell viability and necrosis were tested by CCK-8 (**F** and **J**) and medium LDH release (**G** and **K**) assays, respectively. For the JC-1 assays the representative JC-1 images, integrating both green and red fluorescence images, were presented (same for all Figures). For the Mito-IP assay, CypD-bound p53 and ANT-1 were quantified (**A**). For the cytochrome C release measurement, cytosol cytochrome C (*vs.* Tubulin) was quantified (**C**). Error bars stand for mean ± standard deviation (SD, n=5). “Ctrl” stands for “Mock” control treatment. *p<0.05 *vs.* “Ctrl”. ^#^*p*<0.05 *vs.* cells with “Veh” pretreatment. Each experiment was repeated three times with similar results obtained. Bar=100 μm (**B** and **H**).

In line with the previous findings, we here showed that OGDR stimulation in T-HESC endometrial cells induced programmed necrosis [7, 8]. It was evidenced by cyclophilin-D (CypD)-p53-adenine nucleotide translocator-1 (ANT-1) mitochondrial association (Figure 2A), mitochondrial depolarization (JC-1 green fluorescence accumulation, Figure 2B) and cytosol cytochrome c release (Figure 2C). Furthermore, in OGDR-treated T-HESC cells, significant ROS production was detected by superoxide accumulation (Figure 2D) and increase levels of lipid peroxidation (TBAR intensity, Figure 2E). Importantly, GRh3 pretreatment (for 2h) significantly attenuated OGDR-induced programmed necrosis and oxidative injury in T-HESC cells (Figure 2A–2E).

Functional studies demonstrated that in T-HESC cells OGDR-induced viability (CCK-8 OD value) reduction (Figure 2F) and necrosis (medium LDH release, Figure 2G) were largely attenuated by GRh3 pretreatment. Similarly, GRh3 pretreatment in the primary murine endometrial cells significantly alleviated OGDR-induced mitochondrial depolarization (Figure 2H), superoxide accumulation (Figure 2I), cell viability reduction (Figure 2J) and cell necrosis (LDH release, Figure 2J). These results demonstrated that GRh3 significantly attenuated OGDR-induced oxidative injury and programmed necrosis in endometrial cells. The GRh3 single treatment failed to significantly alter the functions of the endometrial cells (Figure 2A–2K).

### Nrf2 activation is required for GRh3-induced endometrial cell protection against OGDR

To support that Nrf2 activation is required for GRh3-induced endometrial cell protection against OGDR, we utilized genetic methods as previously described [7]. First, two lentiviral shRNAs, with non-overlapping sequences targeting *human Nrf2*, were individually transfected to T-HESC cells. Following selection by puromycin two stable cell lines, namely “shNrf2-a” and “shNrf2-b”, were established. Furthermore, the lenti-CRISPR-Cas9-Nrf2-KO-GFP construct (described in our previous study [7]) was transduced to T-HESC cells. With FACS-mediated sorting, single stable cells were established: named the “ko-Nrf2” cells.

**Figure 3:**
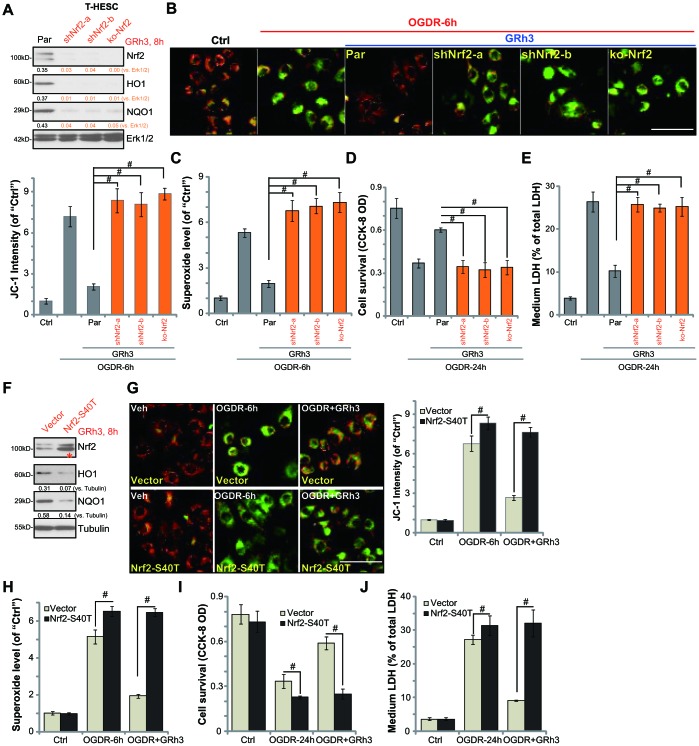
**Nrf2 activation is required for GRh3-induced endometrial cell protection against OGDR.** Stable T-HESC cells, with Nrf2 shRNA (“-a/-b”, different sequences) (**A**–**E**), the CRISPR-Cas9-Nrf2-KO construct (“ko-Nrf2”) (**A**–**E**) or the Nrf2 S40T mutant construct (“Nrf2-S40T”) (**F**–**J**), were treated with GRh3 (10 μM) for 8h; Expression of listed proteins in total cell lysates was shown (**A** and **F**). Cells were pretreated with GRh3 (10 μM) for 2h, followed by OGD (4h)-reoxygenation (“OGDR”) for applied time periods, then the mitochondrial depolarization (JC-1 green fluorescence, B and G) and superoxide contents (**C** and **H**) were tested, with cell viability and necrosis examined by CCK-8 (**D** and **I**) and LDH release (**E** and **J**) assays, respectively. “Pare” stands for the parental control cells (**A**–**E**). “Vector” stands for control cells with empty vector (**F**–**J**). Expression of the listed proteins was quantified, after normalizing to the loading control protein (**A** and **F**). Error bars stand for mean ± standard deviation (SD, n=5). ^#^*p*<0.05. Each experiment was repeated three times with similar results obtained. Bar=100 μm (**B** and **G**).

As shown, the applied Nrf2 shRNA or Nrf2-KO construct resulted in depletion of Nrf2 in T-HESC cells, even with GRh3 stimulation (Figure 3A). Furthermore, GRh3-induced HO1 and NQO1 expression was completely blocked by Nrf2 shRNA or KO (Figure 3A). Significantly, in Nrf2-silenced or Nrf2-KO T-HESC cells, GRh3 pretreatment failed to inhibit OGDR-induced mitochondrial depolarization (JC-1 assay, Figure 3B) and ROS production (testing superoxide contents, Figure 3C). Furthermore, GRh3 was ineffective against OGDR-induced viability (CCK-8 OD) reduction (Figure 3D) and cell death (LDH release, Figure 3E) in Nrf2-depleted T-HESC cells. Therefore, GRh3-mediated T-HESC cell protection against OGDR was abolished by Nrf2 silencing or KO, suggesting that Nrf2 activation is required for GRh3-induced actions in the endometrial cells.

To test whether Nrf2 Ser-40 phosphorylation is the primary mechanism of Nrf2 signaling cascade activation by GRh3, the lentiviral S40T Nrf2 construct (from Dr. Jiang [24]) was transfected to T-HESC cells, with stable cells established following puromycin selection (see Methods). Western blotting results, in Figure 3F, confirmed expression of S40T mutant Nrf2 (“red star”) in the stable cells. GRh3-induced Nrf2 activation, tested by HO1 and NQO1 expression, was largely inhibited by Nrf2 S40T mutation (Figure 3F).

Significantly, in T-HESC cells with Nrf2 S40T mutation, OGDR-induced mitochondrial depolarization (JC-1 assay, Figure 3G) and ROS production (testing superoxide contents, Figure 3H) as well as viability reduction (Figure 3I) and cell death (Figure 3J) were increased. More importantly, GRh3 pretreatment was completely ineffective to protect S40T Nrf2 T-HESC cells from OGDR (Figure 3G–3J). These results suggest that Nrf2 Ser40 phosphorylation is essential for GRh3-induced Nrf2 signaling cascade activation and endometrial cell protection against OGDR.

### Forced activation of Nrf2 by Keap1 KO mimics and nullifies GRh3-induced endometrial cell protection against OGDR

**Figure 4:**
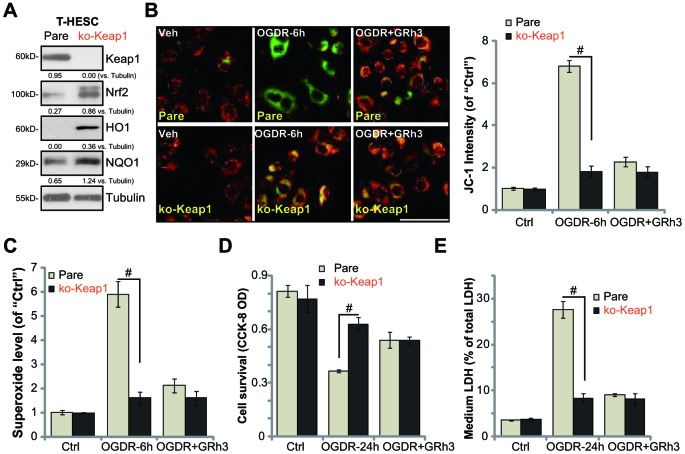
**Forced activation of Nrf2 by Keap1 KO mimics and nullifies GRh3-induced endometrial cell protection against OGDR.** Expression of listed proteins in total cell lysates of stable T-HESC cells with the CRISPR-Cas9-Keap1-KO construct (“ko-Keap1”) or the empty vector (“Vector”) was shown (**A**). Cells were pretreated with GRh3 (10 μM) for 2h, followed by OGD (4h)-reoxygenation (“OGDR”) for applied time periods, then mitochondrial depolarization (JC-1 green intensity, **B**) and ROS production (superoxide contents, (**C**) were tested, with cell viability and necrosis examined by CCK-8 (**D**) and LDH release (**E**) assays respectively. Expression of the listed proteins was quantified, after normalizing to the loading control protein (**A**). Error bars stand for mean ± standard deviation (SD, n=5). ^#^*p*<0.05. Each experiment was repeated three times with similar results obtained. Bar=100 μm (**B**).

Based on the above results, we hypothesized that forced activation of Nrf2 should protect endometrial cells from OGDR, mimicking GRh3’s activity. In order to test the hypothesis, a lentiviral Keap1 KO construct (provided by Dr. Di [25]) was transduced to T-HESC cells, causing complete Keap1 depletion (“ko-Keap1”, Figure 4A). As expected, in Keap1 KO T-HESC cells, Nrf2 protein was accumulated (Figure 4A). Expression of HO1 and NQO1 was significantly increased (Figure 4A). As shown, OGDR-induced mitochondrial depolarization (JC-1 assay, Figure 4B) and superoxide accumulation (Figure 4C) were largely inhibited in the Keap1 KO cells. Significantly, the Keap1 KO T-HESC cells were protected from OGDR, showing significantly decreased viability reduction (Figure 4D) and cell death (Figure 4E) following OGDR treatment. More importantly, GRh3 treatment failed to further protect the Keap1 KO T-HESC cells from OGDR (Figure 4B–4E). Therefore, GRh3 was ineffective in the Keap1 KO cells, further supporting that activation of Nrf2 is required for GRh3-induced endometrial cell protection against OGDR.

## DISCUSSION

Our group [7, 8] and others have shown that OGDR mainly induces programmed necrosis, but not apoptosis in endometrial cells and other human cells [26, 27]. Unlike the passive cell necrosis, programmed necrosis is an active and mitochondria-dependent cell necrosis procedure [26, 27]. Our previous studies have shown that in endometrial cells OGDR induced p53 translocation to mitochondria, forming a complex with mitochondria-localized proteins, ANT-1 and CypD. That will lead to mitochondrial depolarization, mitochondrial permeability transition pore (mPTP) opening, as well as cytochrome C release and ROS production. These signaling events can eventually trigger programmed necrosis, but not apoptosis [8]. In the present study, we show that GRh3 pretreatment potently inhibited OGDR-induced programmed necrosis in the endometrial cells. This might explain its superior endometrial cell-protective ability against OGDR.

The results of this study show that GRh3 induced significant Nrf2 signaling activation in endometrial cells. Nrf2 cascade activation was evidenced by Nrf2 Ser40 phosphorylation and Keap1-Nrf2 disassociation, as well as Nrf2 protein stabilization and nuclear translocation. Moreover, expression of Nrf2-dependent genes, *HO1*, *NQO1* and *GCLC*, was significantly increased in GRh3-treated endometrial cells. Activation of Nrf2 by GRh3 potently attenuated OGDR-induced ROS production and lipid peroxidation. Significantly, Nrf2 activation is required for GRh3-induced actions in endometrial cells. Nrf2 shRNA or KO abolished GRh3-induced anti-oxidative activity and endometrial cell protection against OGDR. Furthermore, forced activation of Nrf2, by Keap1 KO, mimicked and also nullified GRh3-induced endometrial cell protection. Therefore, GRh3-induced endometrial cell protection against OGDR requires Nrf2 activation.

Nrf2 post-transcriptional modification is one key mechanism for Nrf2 signaling cascade activation [28]. Existing studies have shown that Nrf2 phosphorylation at Ser-40 can induced Keap1-Nrf2 disassociation and Nrf2 cascade activation [29]. Our previous study has shown that KGF phosphorylated Nrf2 at Ser40 to promote Nrf2 signaling activation in endometrial cells [7]. Here we found that GRh3 induced Nrf2 Ser40 phosphorylation. Significantly, Nrf2 S40T mutation not only abolished GRh3-induced Nrf2 cascade activation, but also reversed GRh3-induced anti-oxidative activity and endometrial cell protection against OGDR. These results imply that Nrf2 Ser40 phosphorylation should be the key mechanism of Nrf2 cascade activation by GRh3.

One weakness of the study is the lack of *in vivo* studies. Further studies will be needed to test the potential activity of GRh3 in animal models of postpartum hemorrhage. Postpartum hemorrhage is one important contributor of maternal morbidity and mortality. Current treatment options include operational and pharmacologic interventions [1–3]. We propose that GRh3 could be a promising therapeutic option for ischemia-reperfusion-related endometrial disorders.

## CONCLUSIONS

GRh3 protects endometrial cells from OGDR via activation of Nrf2 signaling.

## MATERIALS AND METHODS

### Chemical and reagents

Puromycin and polybrene were obtained from Sigma-Aldrich Chemicals (St Louis, Mo, USA). The cell culture reagents were provided by Gibco BRL (Grand Island, NY, USA). A Nrf2 Ser-40 antibody was from Dr. Jiang [22, 30] at Nanjing Medical University. Other antibodies were obtained from Santa Cruz Biotechnology (Santa Cruz, CA, USA). Lipofectamine 2000 and other transfection reagents were provided by Thermo-Fisher (Shanghai, China).

### Cell culture

T-HESC cells, the immortalized human endometrial cells, were cultured as described previously [8, 31]. The primary culture of murine endometrial (stromal) cells was described early [7, 8]. The protocol of the current study was approved by the Ethics Board of the Affiliated Changzhou No. 2 People’s Hospital of Nanjing Medical University (PI: Hong-bin Xu).

### Cell viability

At 3000 cells per well, T-HESC cells or the primary murine endometrial cells were seeded into 96-well plates. By using cell counting kit-8 (CCK8, Dojindo Laboratories, Kumamoto, Japan) the cell viability was measured [8], with the optical density (OD) absorbance tested at the wavelength of 450 nm.

### Lactate dehydrogenase (LDH) assay

At 5 × 10^4^ cells per well, T-HESC cells or the primary murine endometrial cells were seeded into 12-well plates. Following the applied OGDR treatment, LDH release to the medium, the quantitative measurement of cell necrosis, was tested by a two-step LDH detection kit (Promega, Shanghai, China).

### OGD/reoxygenation (OGDR)

The detailed procedure of OGDR was previously described [8]. In brief, T-HESC cells or the primary murine endometrial cells were placed in an airtight chamber (95% N_2_/5% CO_2_) for 4h (OGD procedure). Thereafter, cells were maintained in the complete medium and re-oxygenated (OGDR) for indicated time periods.

### Western blotting

At 1.5 × 10^5^ cells per well, T-HESC cells or the primary murine endometrial cells were seeded into six-well plates. After the applied OGDR procedure, RIPA lysis buffer (Beyotime Biotechnology Co., Wuxi, Jiangsu, China) was added to obtain total cell lysates. Quantified protein lysates (40 μg per treatment of each lane) were separated by a 10% SDS-PAGE gel, then transferred to a polyvinylidene difluoride (PVDF, Millipore, Shanghai, China) blot [32]. Western blotting analysis and blot data quantification (by using the NIH ImageJ software) were previously described [30, 33]. Assaying of nuclear fraction proteins was also described early [30].

### Co-immunoprecipitation (Co-IP)

The detailed protocols were described previously [34, 35]. After the applied OGDR treatment, RIPA lysis buffer was added to cultured T-HESC cells. For each treatment, 800 μg total cell lysates were incubated with protein G-Sepharose (“Beads”, Sigma, St Louis, Mo, USA) for the pre-clear process. Afterwards, the Keap-1 antibody was added to the lysates overnight at 4 °C. Keap1-immunoprecipitated proteins were then captured by “Beads”, and tested by Western blotting analyses.

### Mitochondrial immunoprecipitation (Mito-IP)

The protocol of Mito-IP was described previously [7, 8]. Briefly, following the applied OGDR treatment, mitochondrial lysates were achieved [34, 35]. The lysates (400 μg of each sample) were pre-cleared and incubated with anti-cyclophilin-D (CypD) antibody (Santa Cruz Biotech, Santa Cruz, CA, USA). CypD-immunoprecipitated proteins were captured and tested by Western blotting analyses.

### Mitochondrial depolarization

At 2 × 10^4^ cells per well, T-HESC cells were seeded into six-well plates. Following the applied OGDR treatment, the mito-dye JC-1 (tetrachloro-tetraethyl-benzimidazolyl carbocyanine iodide, Sigma, St Louis, Mo, USA) assay was performed to test mitochondrial depolarization (“ΔΨ”), using the described protocol [8]. Under a fluorescence spectrophotometer, JC-1 fluorescence intensity was detected at the wavelength of 530 nm. The representative JC-1 images, integrating both green (550 nm) and red (425 nm) fluorescence images, were presented as well.

### Superoxide detection

The endometrial cells, with or without the applied OGDR stimulation, were subjected to the superoxide colorimetric assay (a kit from BioVision, Shanghai, China), examining the cellular superoxide contents. The superoxide detection reagent (50 μL/well) was added for 15 min under the dark, with the superoxide’s absorbance tested at the wavelength of 450 nm [36].

### Lipid peroxidation

As previously described [7, 8], a TBAR (thiobarbituric acid reactive substances) assay was performed to quantify cellular lipid peroxidation levels [37]. At 1.5 × 10^5^ cells per well, T-HESC cells were seeded into six-well plates. Following the applied OGDR treatment, total cell lysates (20 μg of each treatment) were mixed with 20% of acetic acid and TBAR solution. After heating, the mixture was centrifuged, and the red pigment dye in the supernatant was quantified [37]. TBAR values were always normalized to those of untreated control cells.

### Quantitative real time-PCR (qPCR) assay

At 1.5 × 10^5^ cells per well, T-HESC cells or the primary murine endometrial cells were seeded into six-well plates. After the applied treatment, total cellular RNA was extracted by using the TRIzol reagents (Thermo-Fisher Invitrogen, Shanghai, China) [8]. Using an ABI Prism 7900 Fast Real-Time PCR system, qPCR was performed. The product melting temperature was calculated. mRNA primers of listed genes were provided by Dr. Jiang from Nanjing Medical University [21, 30, 38]. A 2^−ΔΔ*C*t^ method was applied for the quantification of targeted mRNAs, using *glyceraldehyde-3-phosphatedehydrogenase*(*GAPDH)* as the internal control.

### Nrf2 shRNA

As previously described [7], two lentiviral Nrf2 shRNAs were purchased from Santa Cruz Biotech (sc-37030-V/”shNrf2-a” and sc-44332-V/”shNrf2-b”). Nrf2 shRNA lentiviral particles were added to cultured T-HESC cells. After 24h, cells were cultured in fresh complete medium, and puromycin (2.0 μg/mL) added to select the stable cells (for 4-5 passages). Over 95% Nrf2 knockdown in the stable cells was verified by Western blotting and qPCR analyses.

### Nrf2 knockout

As previously described [7], the lentiCRISPR-GFP-Nrf2 knockout (KO)-puro construct was transduced to T-HESC cells by Lipofectamine 2000 (Invitrogen, Shanghai, China). The GFP-positive cells were FACS-sorted, and stable cells further selected by puromycin. Nrf2 KO was confirmed by qPCR and Western blotting analyses. Control T-HESC cells were transfected with the empty vector.

### Nrf2 mutation

The S40T dominant negative Nrf2 pSV2 puro Flag plasmid was provided by Dr. Jiang at Nanjing Medical University [21, 22, 24]. At 1 × 105 cells per well, T-HESC cells were seeded into six-well plates. The DN-Nrf2 plasmid or the empty vector was transfected to T-HESC cells using the Lipofectamine 2000 protocol (Invitrogen, Shanghai, China). Stable cells with the S40T Nrf2 or the vector were selected via puromycin.

### Keap1 knockout

The lentiviral Keap1 CRISPR-Cas9 KO construct was provided by Dr. Di from Wannan Medical College [25]. The construct was added to T-HESC cells. After 24h, cells were cultured in fresh complete medium, and puromycin (2.0 μg/mL) added to select the stable cells (for 4-5 passages). Keap1 KO was verified by Western blotting assay in the stable cells.

### Statistical analysis

Data were presented as mean ± standard deviation (SD). Analysis of variance (ANOVA) followed by Dunnett’s post hoc test for multiple comparisons (SPSS 19.0) was performed to test statistical significance. A 2-tailed unpaired T test (Excel 2013, Microsoft) was utilized to test significance between two treatment groups. Values of ***p*** < 0.05 were considered statistically significant.
